# Genome-Wide Association Studies of Mineral Content in Common Bean

**DOI:** 10.3389/fpls.2021.636484

**Published:** 2021-03-05

**Authors:** Jerko Gunjača, Klaudija Carović-Stanko, Boris Lazarević, Monika Vidak, Marko Petek, Zlatko Liber, Zlatko Šatović

**Affiliations:** ^1^Department of Plant Breeding, Genetics and Biometrics, Faculty of Agriculture, University of Zagreb, Zagreb, Croatia; ^2^Centre of Excellence for Biodiversity and Molecular Plant Breeding (CoE CroP-BioDiv), Zagreb, Croatia; ^3^Department of Seed Science and Technology, Faculty of Agriculture, University of Zagreb, Zagreb, Croatia; ^4^Department of Plant Nutrition, Faculty of Agriculture, University of Zagreb, Zagreb, Croatia; ^5^Department of Biology, Faculty of Science, University of Zagreb, Zagreb, Croatia

**Keywords:** *Phaseolus vulgaris* L., landraces, DArTseq, SNP, GWAS, seed mineral content

## Abstract

Micronutrient malnutrition is one of the main public health problems in many parts of the world. This problem raises the attention of all valuable sources of micronutrients for the human diet, such as common bean (*Phaseolus vulgaris* L.). In this research, a panel of 174 accessions representing Croatian common bean landraces was phenotyped for seed content of eight nutrients (N, P, K, Ca, Mg, Fe, Zn, and Mn), and genotyped using 6,311 high-quality DArTseq-derived SNP markers. A genome-wide association study (GWAS) was then performed to identify new genetic sources for improving seed mineral content. Twenty-two quantitative trait nucleotides (QTN) associated with seed nitrogen content were discovered on chromosomes Pv01, Pv02, Pv03, Pv05, Pv07, Pv08, and Pv10. Five QTNs were associated with seed phosphorus content, four on chromosome Pv07, and one on Pv08. A single significant QTN was found for seed calcium content on chromosome Pv09 and for seed magnesium content on Pv08. Finally, two QTNs associated with seed zinc content were identified on Pv06 while no QTNs were found to be associated with seed potassium, iron, or manganese content. Our results demonstrate the utility of GWAS for understanding the genetic architecture of seed nutritional traits in common bean and have utility for future enrichment of seed with macro– and micronutrients through genomics-assisted breeding.

## Introduction

Micronutrient malnutrition, known as “hidden hunger,” particularly the lack of minerals such as Fe and Zn, is the main global nutritional problem (Hirschi, 2009; Diepenbrock and Gore, 2015; Semba, 2016; Yeken et al., 2018), due to great importance of micronutrients in fundamental biological functions (Tapiero et al., 2003). Common bean (*Phaseolus vulgaris* L.) is a species of great interest for human diet worldwide, gaining attention as functional food offering benefits for human health (Câmara et al., 2013). It provides macro- and micronutrients (especially Fe and Zn) and because of high protein content, together with other pulses common bean is known as poor man’s meat (Gouveia et al., 2014; Mahajan et al., 2017; Nwadike et al., 2018). The nutritional composition of common bean landraces depends on factors like origin, genotype and environmental conditions (Gouveia et al., 2014). Moreover, researches that have analyzed the genetic control of seed composition were mainly focused on minerals such as iron, phosphorus and zinc (Blair et al., 2009; Cichy et al., 2009), since they are among the most important nutritional deficiencies in humans.

The availability of molecular markers has enabled the determination of the origin and diversity of populations, as well as the elucidation of the genetic basis of important complex agronomic traits with increased resolution (Gioia et al., 2013; Chávez-Servia et al., 2016; Valdisser et al., 2017). For this purpose, microsatellite markers have been the most widely used markers over the past decade (Razvi et al., 2017; Valdisser et al., 2017). In recent years, single nucleotide polymorphism (SNP) markers have been developed and increasingly used for genetic and evolutionary studies, analysis of genome structure, genetic diversity analysis, genome-wide association mapping and integration of genetic maps representing a useful tool for plant breeding purposes (Goretti et al., 2014; Villordo-Pineda et al., 2015; Nemli et al., 2017; Valdisser et al., 2017). Diversity Arrays Technology (DArTseq), based on genome complexity reduction and SNP detection through hybridization of PCR fragments (Jaccoud et al., 2001), has been successfully used for the construction of dense linkage maps and quantitative trait locus QTL analysis, genome-wide association studies (GWAS) and genetic diversity studies (Valdisser et al., 2017).

Over the last decade, genome-wide association study (GWAS) has become a popular approach for studying traits of agricultural importance and has gained popularity particularly for screening a great number of accessions to gain insight into understanding the genetic basis of complex traits (Yu et al., 2006). In common bean, GWAS has been used to identify genes controlling traits such as disease resistance (Shi et al., 2011; Perseguini et al., 2016; Zuiderveen et al., 2016; Tock et al., 2017, Fritsche-Neto et al., 2019), drought-tolerance related traits (Galeano et al., 2012; Hoyos-Villegas et al., 2017), agronomic traits in general (Nemli et al., 2014; Kamfwa et al., 2015b; Moghaddam et al., 2016; Ates et al., 2018; Nascimento et al., 2018; Resende et al., 2018), nitrogen fixation (Kamfwa et al., 2015a), cooking time (Cichy et al., 2015), flooding tolerance (Soltani et al., 2017; Soltani et al., 2018), content of micronutrients (Mahajan et al., 2017; Katuuramu et al., 2018; Myers et al., 2019; Diaz et al., 2020; Erdogmus et al., 2020), and pod shattering (Rau et al., 2019).

The basic goal of GWAS is to detect markers that are either associated with a trait of interest directly or are in linkage disequilibrium (LD: non-random association of alleles at different loci in a given population) with a quantitative trait locus (QTL) that controls it. Cited GWAS studies on content of micronutrients detected numerous QTLs associated with Fe, Ca, Zn and Mn content, located an all chromosomes. Most of them represent minor genes, usually explaining around 10% or less of total phenotypic variation, with only a few exceptions. Earlier studies employing classical QTL analysis summarily detected a large number of QTLs; some of them were associated with major genes, but they were usually population or environment specific (Blair et al., 2009; Cichy et al., 2009; Blair et al., 2010, 2011). Pooling together the populations from different studies, meta-analysis resulted in the reduction of the original set of 87 detected QTLs into a set of 12 meta-QTLs, two specific for iron and zinc, and eight common for both minerals (Izquierdo et al., 2018). All discovered QTLs provide promising potential for use in plant breeding programs targeted at mineral biofortification, such as HarvestPlus (Pfeiffer and McClafferty, 2007). Newly developed biofortified cultivars exhibit potential for improving the iron status in iron-deficient individuals (Tako et al., 2015; Haas et al., 2016).

Reviewing the GWAS studies in common bean, it is possible to notice that many differences exist in applied strategy, regards the methodology in general, as well as in choices made at the different steps of the process. The markers which are in LD with QTLs controlling the analyzed trait cannot be straightforwardly identified because besides the physical linkage, LD can also be created by the genetic relatedness between individuals and/or population structure. These factors can extend LD over larger chromosomal regions, thus increasing the number of spurious associations that are most likely just false positives. This can be illustrated by the difference between uncorrected and kinship/structure corrected measures of LD (*r*^2^). While uncorrected *r*^2^ indicates strong LD even for the SNPs located on the opposite ends of the chromosome (Valdisser et al., 2017; Resende et al., 2018; Diniz et al., 2019), bias-corrected measures indicate LD decay of *r*^2^ to 0.1 at distances of approximately 250 kbp (Diaz et al., 2020), 400 kbp (Valdisser et al., 2017), 700 kbp (Resende et al., 2018), or up to 1 Mbp (Diniz et al., 2019). Therefore, the prevailing method of detecting marker-trait associations is based on a mixed linear model (MLM) proposed by Yu et al. (2006). It is also known as K + Q model because includes the fixed effect of population structure (Q) and random effect of kinship (K), and it is implemented in software packages such as TASSEL (Bradbury et al., 2007) and GAPIT (Lipka et al., 2012), providing different options for kinship/structure correction. In the next step, raw *p*-values were subjected to multiple testing adjustment in order to remove false positives. Some authors prefer the most stringent method of Bonferroni (Galeano et al., 2012; Kamfwa et al., 2015b; Zuiderveen et al., 2016), the others more relaxed variants of false discovery rate (FDR) adjustment (Cichy et al., 2009; Ates et al., 2018; Katuuramu et al., 2018) or using empirical distribution created by bootstrap to determine the cutoff point (Moghaddam et al., 2016; Soltani et al., 2018). Two studies (Moghaddam et al., 2016; Rau et al., 2019) combined described single-locus approach with the multilocus mixed model (MLMM) of Segura et al. (2012). MLMM is based on the same Q + K model but fitted in the stepwise procedure, adding or excluding a marker as a fixed effect (cofactor) in the model at each step.

In the present study, a panel of 174 accessions representing Croatian common bean landraces was used for GWAS based on DArTseq-derived SNP markers with the aim of identifying quantitative trait nucleotides (QTNs) associated with variation in seed content of eight nutrients (N, P, K, Ca, Mg, Fe, Zn, and Mn). The secondary aim of the study was to compare different methodology options and identify their possible pitfalls and shortcomings.

## Materials and Methods

### Plant Material and Mineral Content Assessment

The study was performed using 174 accessions representing the most commonly used Croatian landraces of common bean (Carović-Stanko et al., 2017) held at the University of Zagreb Faculty of Agriculture, Department of Seed Science and Technology. The list of accessions with their passport data, phaseolin type and cluster membership is given in Supplementary Table 1. Phenotypic data on mineral content were drawn from an earlier study (Palèić et al., 2018) analyzing nitrogen (N), phosphorus (K), potassium (K), calcium (Ca), magnesium (Mg), iron (Fe), zinc (Zn), and manganese (Mn) variability in a broader panel of 226 accessions of the collection.

### Genotyping and Data Preparation

Genotyping was carried out using microsatellite and DArTseq-derived SNP markers. Twenty-six microsatellite markers yielding a total of 135 alleles in the panel consisting of 174 common bean accessions were used to infer the population structure using STRUCTURE 2.3.3 software (Pritchard et al., 2000) as described in Carović-Stanko et al. (2017). Phaseolin type of each accession was determined by amplification of phaseolin sequences (Kami et al., 1995) as described in Carović-Stanko et al. (2017). DArTseq analysis was performed by Diversity Arrays Technology Pty Ltd., Bruce, Australia^1^. The quality of DArTseq-derived SNP markers was determined by the parameters ‘reproducibility’ (percentage of technical replicate pairs scoring identically for a given marker), ‘call-rate’ (percentage of samples for which a given marker was scored) and ‘MAF’ (minor-allele frequency) (Wenzl et al., 2004). Marker sequences were aligned against the reference genome of *Phaseolus vulgaris* (Schmutz et al., 2014) using BLASTN (Zhang et al., 2000). Final SNP data quality control was performed by excluding all SNPs with MAF < 0.05 and all SNPs with >0.05 heterozygotes, resulting in the final set of 6,311 high-quality DArTseq-derived SNPs. The missing SNP data were imputed by using Beagle 5.1 genotype imputation method (Browning et al., 2018). The imputed data set was then used to construct a kinship matrix by applying four methods implemented in TASSEL 5 software (Bradbury et al., 2007): (1) centered IBS (Endelman and Jannink, 2012), (2) normalized IBS (Yang et al., 2011), (3) dominance centered IBS (Muñoz et al., 2014), and (4) dominance normalized IBS (Zhu et al., 2015). Additionally, we used the corrected relatedness matrix as proposed by Diniz et al. (2019).

### Linkage Disequilibrium

Non-random association between alleles at different loci was measured by *r*^2^. Besides straightforward *r*^2^, corrected measures designed to remove the bias caused by population structure (*r*_*S*_^2^), kinship (*r*_*V*_^2^) and both (*r*_*VS*_^2^) were also estimated (Mangin et al., 2012). Both measures involving kinship correction were estimated using five different kinship matrices described above. In order to visualize LD decay as a function of distance, all measures were fitted to Hill and Weir model (Hill and Weir, 1988).

### Genome-Wide Association Study (GWAS)

Before carrying out the GWAS, missing phenotypic data were imputed using PHENIX method as implemented in the eponymous R package (Dahl et al., 2016). Prior to imputation, outliers were removed using the “trim” option in “phenix” (trim.sds = 1.96). GWAS was performed using both single (Yu et al., 2006) and multi-locus model approaches (Segura et al., 2012). Mixed linear models fitted in both cases included corrections for population structure and genetic relatedness (Q and K matrices). Population membership estimates were derived from microsatellite data using STRUCTURE (Pritchard et al., 2000) and the centered IBS method was used for adjustment of genetic relatedness. Single locus models were fitted in TASSEL 5 while the multi-locus models were employed in R package MLMM (Segura et al., 2012). After fitting single-locus models in TASSEL, the resulting raw *p*-values were subjected to multiple testing adjustment. In order to score for more potential marker-trait associations, Storey’s FDR approach was used instead of frequently applied Bonferroni correction (which tends to be too conservative). Row *p*-values from TASSEL were converted into Storey’s *q*-values using R package “qvalue” (Storey et al., 2020), and *q*-value of 0.2 was selected as the significance threshold. Distributions of *p*-values from TASSEL fits across the genome were visualized by Manhattan plots created using R package “CMplot” (Yin et al., 2020). Before creating Manhattan plots for all traits with significant SNPs, for each trait, an approximate threshold was calculated as *p*-value of a hypothetical SNP that would have a *q*-value of 0.2. A similar approximate significance threshold was created for MLMM – *p*-values from step zero were used to estimate *p-*value of hypothetic SNP that would have a *q*-value of approximately 0.2. The distribution of alleles across subpopulations for QTNs was visually inspected by creating violin plots.

## Results

### Genotyping and Data Preparation

In concordance with the results described by Carović-Stanko et al. (2017), the STRUCTURE analysis based on 26 microsatellite loci identified *K* = 2 as the most likely number of clusters (Δ*K* = 20,533.24) assigning the accessions of Mesoamerican origin (phaseolin type I; “S”) to cluster A, while the accessions of Andean origin (phaseolin type II or III) formed the cluster B. At *K* = 3 (Δ*K* = 1,935.93), cluster B defined for *K* = 2 split up into two clusters separating the great majority of accessions of phaseolin type II (“H” or “C”) from those having phaseolin type III (“T”). At *K* = 3, 48 accessions (27.59%) belonged to cluster A, 29 (16.67%) to cluster B_1_, and 80 (45.96%) to cluster B_2_. For 17 accessions (9.77%), membership probabilities were lower than 75% in any of the clusters and were thus considered as “mixed origin.” Phaseolin type and cluster membership of each accession are given in Supplementary Table 1. The *Q*-values of each accession obtained at *K* = 3 were used for the control of genetic background in GWAS.

Out of 17,514 polymorphic markers 8,092 (46%) had high scoring reproducibility (>0.95), high call-rate (>0.90) and minor allele frequency (MAF) higher than 5%. From 8,092 SNP sequences, 6,599 (82%) high-quality SNPs were aligned to 11 chromosomes of common bean. The average number of SNPs per chromosome was 599.91, ranging from 403 on chromosome 4 to 834 on chromosome 2. The mean number of SNPs per Mbp was 12.85 or, on average, one SNP every 77,828 base pairs. Two hundred and eighty-eight SNPs for which more than 5% of accessions were heterozygous were removed for further analysis, and missing data were imputed for the remaining 6,311 SNPs (Supplementary Table 2).

### Linkage Disequilibrium

**FIGURE 1:**
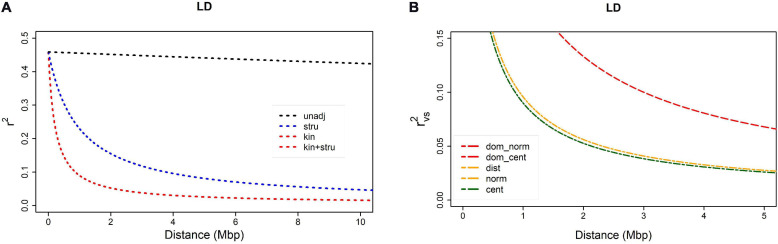
Linkage disequilibrium (LD) decay as a function of distance between SNPs within a chromosome: **(A)** comparison of four *r*^2^ measures – *r*_*v*_^2^ and *r*_*vs*_^2^ are based on centered IBS kinship: (1) *r*^2^ = unadj, (2) *r*_*s*_^2^ = stru, (3) *r*_*v*_^2^ = kin, (4) *r*_*vs*_^2^ = kin + stru); **(B)** comparison of *r*_*vs*_^2^ based on five different kinship matrices: (1) dominance normalized, (2) dominance centered, (3) distance, (4) normalized, (5) centered.

Linkage disequilibrium, non-independence of alleles at different loci was assessed as the squared correlation between loci (*r*^2^). Bias caused by relatedness and/or population structure was removed by adjusting *r*^2^: (a) using kinship estimates (*r*_*v*_^2^), (b) using *Q*-values obtained by STRUCTURE (*r*_*s*_^2^), or (c) using both (*r*_*vs*_^2^). Fitted model for non-adjusted estimate *r*^2^ (Figure 1A) showed that LD decay was barely visible within 0–10 Mbp distance range, remaining above 0.3 even for pairs of loci at the opposite ends of a chromosome. On the contrary, when adjusted for population structure, LD decayed to 0.1 at an approximate distance of 4 Mbp. The *r*^2^ value of 0.1 could then be taken as an arbitrary threshold for comparison of different measures. Bias caused by relatedness was even stronger, and there was almost no difference between adjustment for kinship only and for kinship and population structure, both reaching LD decay threshold (*r*^2^ = 0.1) at approximately 1 Mbp. Both measures, *r*_*v*_^2^ and *r*_*vs*_^2^, shown on Figure 1A were based on a centered identity-by-state (IBS) kinship matrix. The *r*_*vs*_^2^ based on centered IBS was also shown in Figure 1B, in which it was compared to *r*_*vs*_^2^ values based on four other kinship matrices. Estimation curves for normalized IBS and distance-based kinships were completely overlapped, and the difference between them in comparison to centralized IBS was visible only due to magnification achieved by reducing both axes to approximately 1/10 of their total range. Two dominance-based kinship curves were completely overlapped as well, but characterized with lower decay rate, reaching 0.1 threshold at the distance of approximately 3 Mbp. The results suggested that adjustment for relatedness preferably using a centralized IBS kinship matrix was a necessary requirement for GWAS.

### Genome-Wide Association Study (GWAS)

Before carrying out the GWAS, missing phenotypic data were imputed using a centered IBS kinship matrix. Prior to imputation, outliers (>1.96 standard deviations) were trimmed, thus removing from one to ten the most extreme data points per trait. Descriptive statistics for all minerals based on imputed data set are given in Table 1.

**TABLE 1:** Descriptive statistics for seed mineral content in 174 Croatian common bean accessions.

Trait	Unit^*a*^	Mean	Standard deviation	Range
Nitrogen (N)	% DW	3.46	0.35	2.79–4.19
Phosphorus (P)	% DW	0.52	0.05	0.41–0.64
Potassium (K)	% DW	1.43	0.12	1.20–1.83
Calcium (Ca)	% DW	0.34	0.09	0.20–0.65
Magnesium (Mg)	% DW	0.18	0.02	0.13–0.24
Iron (Fe)	mg⋅kg^–1^ DW	71.53	8.43	49.93–93.28
Zinc (Zn)	mg⋅kg^–1^ DW	27.23	3.49	19.32–35.65
Manganese (Mn)	mg⋅kg^–1^ DW	15.98	2.34	10.94–21.48

*^*a*^DW, dry weight.*

**FIGURE 2:**
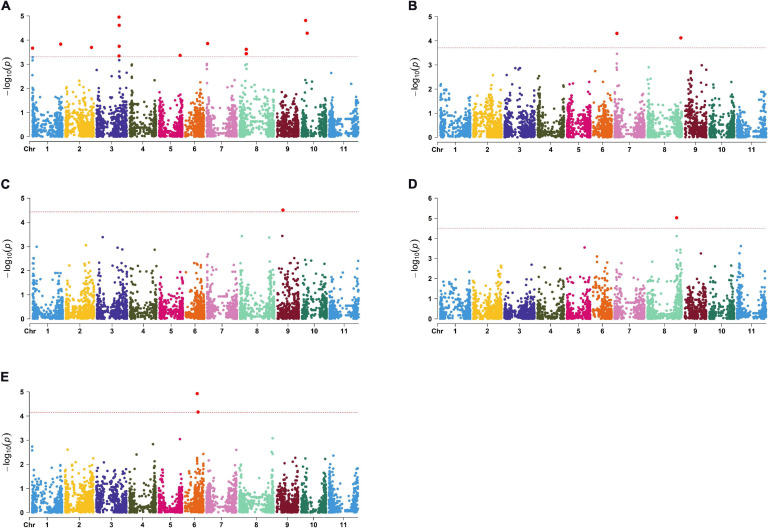
Manhattan plots for significant markers detected by TASSEL: **(A)** N; **(B)** P; **(C)** Ca; **(D)** Mg; **(E)** Zn.

The largest number of significant SNPs considered as QTN was discovered for N (Figure 2A); in total there were 22 significant QTNs on seven chromosomes. Among them, the highest observed -log_10_(*p*) peaks were observed at two pairs of QTNs located on chromosome 3 (Pv03) and chromosome 10 (Pv10), both explaining 7% of total phenotypic variation. Five QTNs were associated with P: four on chromosome 7 (Pv07) and one on chromosome 8 (Pv08) (Figure 2B), explaining 8–9% of variation. A single significant QTN was found for Ca on chromosome 9 (Pv09) (Figure 2C) and Mg on Pv08 (Figure 2D), explaining 9 and 13% of variation, respectively. Finally, two QTNs associated with Zn were located 1.4 Mbp apart from each other on Pv06 (Figure 2E), explaining 8 and 10% of variation. No QTNs were found to be associated with K, Fe, and Mn.

As expected, a multi-locus model fitting in MLMM resulted in much less marker-trait association discoveries. Out of 22 QTNs associated with N by TASSEL, only two were confirmed by MLMM: one out of four on Pv01 and the first of the two QTNs on Pv10. Similarly, only one QTN out of four found by TASSEL on Pv07 was associated with P. An additional discovery by MLMM is QTN associated with N on the Pv05, not previously detected by TASSEL.

**FIGURE 3:**
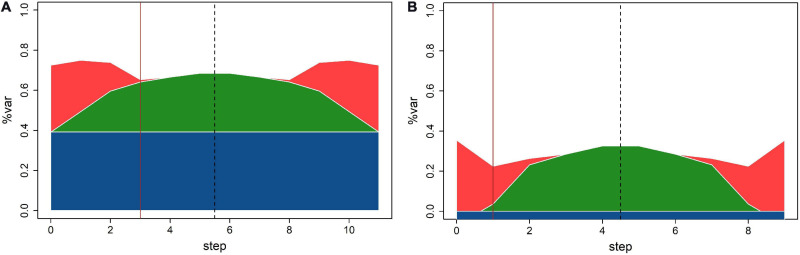
Variability breakdown at different MLMM steps for **(A)** N; **(B)** P. Structure (blue), SNPs (green), kinship (red), and error (white). Solid vertical line designates optimal step.

Regarding the relationships between sizes of different variance components estimates obtained by MLMM, comparison of the residual sum of squares (RSS) plots for N and P (Figure 3) could be summarized in two key points: (1) population structure explained 40% of total N variation and 0% of total P variation; (2) error variation was of similar size as genetic variation in N and twice as large in P. Consequentially, despite similar relative size of genetic variation for N and P, MLMM detected three QTNs with *p*-values below the threshold for N, and only one for P.

The summary of all marker-trait associations detected either by TASSEL or MLMM is given in Table 2. The highest overall explanatory power was recorded for the QTN Mg_8, explaining 13% of the total phenotypic variability for Mg. Instead of an individual *R*^2^ value for each marker, only a cumulative value for the full set of markers could be extracted from MLMM output. The associated markers were distributed over the whole genome, except for the chromosomes Pv04 and Pv11; N was the trait with the largest number of discovered associations, but individual effects of markers were lower than for other traits. When the TASSEL discovered a sequence of QTNs within up to 0.3 Mbp distance range, MLMM would likely retain just one of them. Finally, most of the markers were positioned closer to the chromosome ends, and just a few closer to the centromeric region.

**TABLE 2:** Positions of the quantitative trait nucleotides (QTNs) associated with seed mineral content in common been.

Trait	QTN	Chr.	Position (bp)	Method of detection	−log_10_(*p*)^*a*^	*R*^2^	MAF^*b*^	SNP^*c*^	Additivity^*d*^
N	N_1.1	Pv01	436,239	TASSEL	3.66	0.05	0.11	G/T	0.15
	N_1.2	Pv01	436,255	TASSEL/MLMM	3.16	0.06	0.12	A/G	0.15
	N_1.3	Pv01	459,311	TASSEL	3.29	0.06	0.12	C/T	0.15
	N_1.4	Pv01	49,116,667	TASSEL	3.83	0.06	0.24	A/T	0.06
	N_2	Pv02	45,348,786	TASSEL	3.69	0.07	0.17	G/A	−0.31
	N_3.1	Pv03	38,974,600	TASSEL	4.95	0.07	0.07	A/T	0.23
	N_3.2	Pv03	39,046,040	TASSEL	3.34	0.05	0.22	T/C	−0.20
	N_3.3	Pv03	39,187,621	TASSEL	3.17	0.04	0.21	A/G	−0.20
	N_3.4	Pv03	39,194,457	TASSEL	3.17	0.04	0.21	G/T	−0.20
	N_3.5	Pv03	39,225,372	TASSEL	3.74	0.06	0.08	A/T	0.23
	N_3.6	Pv03	39,225,437	TASSEL	3.17	0.04	0.21	G/A	−0.20
	N_3.7	Pv03	39,235,479	TASSEL	3.17	0.04	0.21	C/T	−0.20
	N_3.8	Pv03	39,249,094	TASSEL	3.17	0.04	0.21	T/G	−0.20
	N_3.9	Pv03	39,252,941	TASSEL	4.61	0.06	0.07	A/G	0.21
	N_5.1	Pv05	877,194	MLMM	4.64	−^*e*^	0.40	C/T	0.06
	N_5.2	Pv05	36,788,205	TASSEL	3.37	0.05	0.28	C/T	0.06
	N_7	Pv07	1,605,873	TASSEL	3.86	0.06	0.07	G/A	0.25
	N_8.1	Pv08	11,652,741	TASSEL	3.43	0.06	0.26	T/C	−0.23
	N_8.2	Pv08	11,773,516	TASSEL	3.44	0.05	0.09	A/T	−0.02
	N_8.3	Pv08	11,864,133	TASSEL	3.62	0.05	0.25	T/A	−0.22
	N_8.4	Pv08	11,977,715	TASSEL	3.44	0.05	0.09	C/T	−0.02
	N_10.1	Pv10	7,465,267	TASSEL/MLMM	4.81	0.07	0.15	A/T	−0.24
	N_10.2	Pv10	10,200,728	TASSEL	4.28	0.06	0.15	A/T	−0.28
P	P_7.1	Pv07	3,864,210	TASSEL/MLMM	4.30	0.09^*f*^	0.19	G/C	0.05
	P_7.2	Pv07	3,888,203	TASSEL	4.30	0.09	0.19	C/A	0.05
	P_7.3	Pv07	3,894,237	TASSEL	4.30	0.09	0.19	T/C	0.05
	P_7.4	Pv07	4,076,469	TASSEL	4.30	0.09	0.19	G/A	0.05
	P_8	Pv08	58,013,431	TASSEL	4.12	0.08	0.22	C/G	−0.02
Ca	Ca_9	Pv09	9,978,327	TASSEL	4.50	0.09	0.05	C/G	−0.13
Mg	Mg_8	Pv08	50,916,423	TASSEL	5.02	0.13	0.15	G/T	0.02
Zn	Zn_6.1	Pv06	21,113,843	TASSEL	4.93	0.10	0.45	A/G	2.43
	Zn_6.2	Pv06	22,539,825	TASSEL	4.16	0.08	0.41	A/T	−2.17

*^*a*^All p-values are reported from F-test from TASSEL, except for N_5.1 (t-test from MLMM). ^*b*^Minor allele frequency. ^*c*^Allelic substitution at SNP locus. ^*d*^The effect of allelic substitution at SNP locus (difference between two homozygote classes). ^*e*^R^2^ value for optimal MLMM model including N_1.2, N_5.1 and N_10.1 is equal to 0.25. ^*f*^R^2^ value for P_7.1 in MLMM is equal to 0.11.*

**FIGURE 4:**
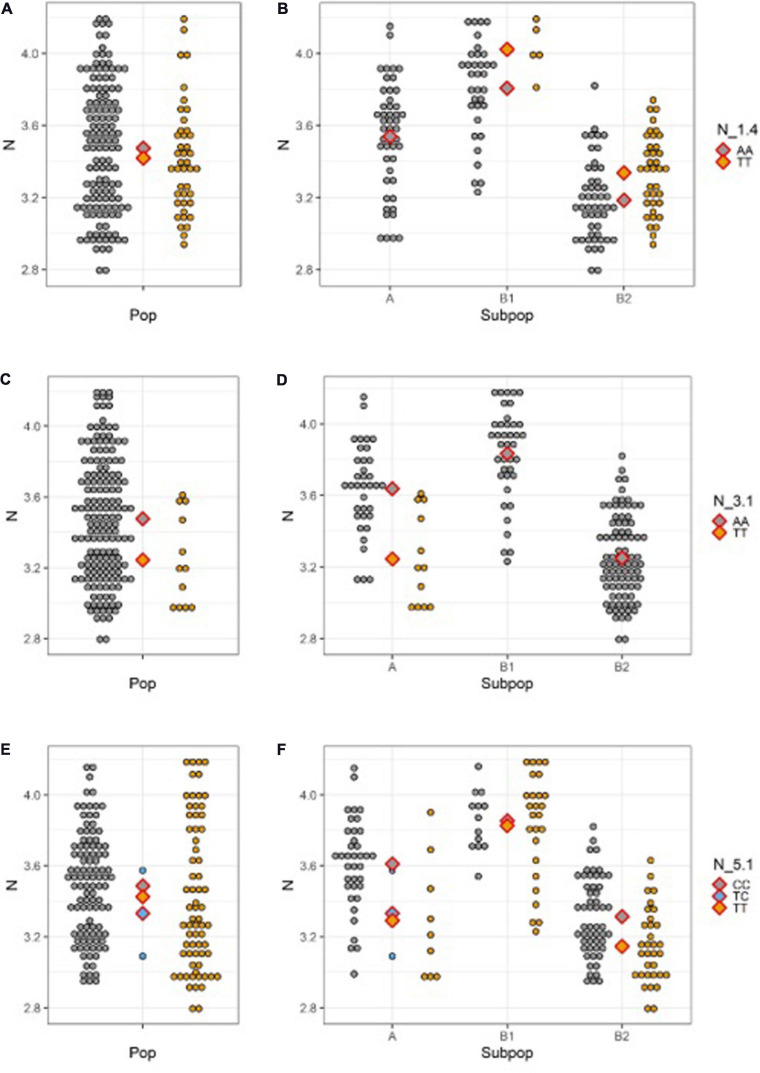
N seed content distribution for different allele classes across the whole population (left) and within subpopulations (right; A Mesoamerican; B1 Andean; B2 Andean) for: **(A,B)** N_1.4; **(C,D)** N_3.1; **(E,F)** N_5.1. Diamonds designate population/subpopulation means for reference allele homozygotes (gray), SNP homozygotes (yellow), and heterozygotes (blue).

Strong effect of population structure in N, clearly visible on Figure 3, deserve to be further elucidated in conjunction with the effect of allele substitution at QTN sites. Figure 4 shows N content distribution in different allele classes for QTNs N_1.4 (a-b), N_3.1 (c-d), and N_5.1 (e-f), across whole population (a, c, e) and within subpopulations (b, d, f). Reference allele for all QTNs was always present in all subpopulations and mean N content of individuals carrying reference allele in the subpopulation A (Mesoamerican origin) is always somewhere in between means of the subpopulations B1 and B2 (Andean origin). There are the three possible scenarios for the distribution of the SNP allele. It could be present only in subpopulations of Andean origin (Figure 4B), but its positive effect observable in both B1 and B2 almost disappeared at whole population level, being masked by the effect of population structure (Figure 4A). In the second scenario, SNP allele is present only in subpopulation A (Mesoamerican) expressing a clear negative effect (Figure 4D), that gets shrunken by the effect of the population structure (Figure 4C). Finally, if the SNP allele is present in all subpopulations, its effect varies from one subpopulation to another (Figure 4F), to became almost invisible at the level of whole population (Figure 4E).

## Discussion

### Genetic Structure and Seed Mineral Content in Common Bean

Concerning the origin of analyzed common bean accessions, we performed a model-based cluster analysis based on microsatellite markers which revealed the presence of three clusters in nearly complete congruence with the results of phaseolin type analysis. The results are consistent with previous studies showing that the European germplasm originates also from Mesoamerican and Andean gene pools, where the Andean is more prevalent (Bellucci et al., 2014). Similar results were obtained for Portuguese germplasm (Leitão et al., 2017) and the germplasm originating from countries neighboring Croatia (Bosnia and Herzegovina: 60% of the accessions of Andean origin; Serbia: 63%; Slovenia: 67%) (Maras et al., 2015).

As one of the requirements for successful GWAS, the presence of a reasonable amount of phenotypic diversity amongst genotypes in the study panel was already established in an earlier study (Palèić et al., 2018). For most of the analyzed traits, the amount of present phenotypic variability was either comparable or slightly narrower (especially if they were comprised of the accessions from regions located close to the center of origin) than variability reported for other collections. Furthermore, Croatian accessions of Mesoamerican origin had superior seed mineral content compared to those of Andean origin which was in congruence with the research of Ribeiro et al. (2012), except for iron, which is congruent to results reported by Beebe et al. (2000) and Islam et al. (2002).

### Linkage Disequilibrium

The major issue in GWAS is to separate the true signal of marker-gene association from a plethora of false signals created by population structure and relatedness of individuals. The impact of kinship and population structure on LD estimates in common beans has been extensively discussed in a recent study by Diniz et al. (2019) that served as a model for designing the methodology of the present study. Despite all the differences in genetic composition and origin of studied populations (Croatian landraces vs. composite panel consisting of commercial cultivars, breeding lines, recombinant inbred lines and landraces) LD estimates from the present study have all the hallmarks of results reported by Diniz et al. (2019). The extent of bias caused by kinship and structure on LD estimates is quite similar: both studies reveal that more bias is introduced by kinship than by structure as there is only a negligible difference between *r*_*v*_^2^ and *r*_*vs*_^2^, and finally, as estimated by *r*_*vs*_^2^, LD decayed to 0.1 at a distance of approximately 1 Mbp. In addition to the conclusions of Diniz et al. (2019), the present study revealed that there is no essential difference between distance-based and centered or normalized IBS kinship estimate, as well as that all three of them outperformed both dominance-based kinship estimates. Almost identical performance of *r*_*v*_^2^ and *r*_*vs*_^2^ measures support Astle and Balding (2009) opinion that adjustment for kinship already contains the adjustment for population structure as well. Several authors have tried to resolve this issue through different modifications of the kinship matrix in order to remove information already contained in the model as fixed effects of population structure. So far, there is no consensus regards this matter, and as Gianola et al. (2016) concluded, “it is impossible to answer unambiguously the question of which approach is best.”

### GWAS Methodology

There are two possible reasons for the discrepancy in the number of marker-trait associations identified by TASSEL and MLMM: (1) stepwise reduction of available genetic variability in MLMM; (2) the use of different methods for estimation of genetic and error variance.

Stepwise reduction of available genetic variability in MLMM: Single locus model used in TASSEL explores the entire genetic variability for fitting each SNP, while the multilocus model (MLMM) reduces available genetic variability for the next step by fixing selected SNP at each fitting step. At the final forward step, the genetic variability is completely exhausted; MLMM stops and runs the backward part of the stepwise fitting, as illustrated by RSS plots in Figure 3. The largest potential number of discoveries is equal to the number of steps, but the number of actual discoveries is equal to the ordinal number of the optimal step. Search for the optimal step is based on the selected threshold value, and it is aimed at finding the last (i.e., first) step in which all *p*-values for fixed markers are below the threshold.

The use of different methods for estimation of genetic and error variance in TASSEL and MLMM: Stepwise reduction of available genetic variability in MLMM: it is not possible to make a straightforward comparison of variance estimates from TASSEL and MLMM, because MLMM output provides only relative values of genetic and error variance, used to create RSS plots on Figure 3. At each step MLMM reports the value of “pseudo-heritability,” the ratio between genetic and sum of genetic and error variance. E.g., this estimate at step 0 for *N* is equal to 0.548, thus substantially larger than 0.377, the value of equivalent ratio calculated using the TASSEL null model variance estimates.

Furthermore, as the results of the TASSEL analysis were obtained by using the option to re-estimate variance estimates for each marker fit, the analysis was redone using the timesaving P3D (“population parameters previously determined”) option. P3D approach uses null model estimates for all marker fits (Zhang et al., 2010). Despite the perfect correlation between the *p* values obtained by the two options (“re-estimate” vs. P3D), the results were in disagreement in terms of the number of discoveries. Namely, using the selected threshold of *q* = 0.2, the analysis using P3D option detected no significant SNPs, because the smallest estimated *q*-value was as high as 0.25. It is likely that this outcome is related to panel size as well as the number of markers.

Application of stringent methods for multiple testing adjustment in the present study would result in no potential QTN finding, thus turning all of them into false negatives. Using a more relaxed approach by selecting Storey’s *q*-value of 0.2 as the cutoff point yielded the reported set of QTNs. It comes with the cost of 20% false positives, i.e., when the analysis of *N* content in TASSEL detected 22 marker-trait associations, 5 of them were actually false. Although other common bean studies used lower FDR cutoff values of 0.01–0.10 (Hoyos-Villegas et al., 2017; Ates et al., 2018; Katuuramu et al., 2018), it is not unusual to find recently published GWAS analyses in some other crops with *q*-value threshold of 0.2 (e.g., Muqaddasi et al., 2017; Novakazi et al., 2019). As Storey and Tibshirani (2003) have pointed out: “because significant features will likely undergo some subsequent biological verification, a *q*-value threshold can be phrased in practical terms as the proportion of significant features that turn out to be false leads”; significant QTNs can be treated as merely input values for further evaluation such as functional annotation.

### QTN Discoveries

There are only few published GWAS analyses of nutrient content in common bean seed (that we are aware of) Two QTNs associated with seed iron content found on chromosome 6 by Diaz et al. (2020) are different from three QTNs associated with bioavailable iron found on the same chromosome by Katuuramu et al. (2018), along with two others on chromosomes 7 and 11. Zinc QTNs were found on chromosomes 6 (present study), 7 (Katuuramu et al., 2018), and 8 (Diaz et al., 2020); manganese QTNs on chromosomes 2, 3, 5, 8, and 11 (Erdogmus et al., 2020). The most abundant were calcium and nitrogen QTNs: for Ca they were found on chromosomes 1, 2, 3, 4, 8, 9, 10, and 11 (Katuuramu et al., 2018; Erdogmus et al., 2020, present study); for N on chromosomes 1, 2, 3, 5, 7, 8, 9, and 10 (Kamfwa et al., 2015a; present study). In addition to this, five phosphorus and a single magnesium QTN were discovered in the present study. According to the available information for their positions, there are no common QTNs detected in more than one study. It could be added, more as a curiosity, that QTN for nitrogen derived from atmosphere detected on chromosome 7 (at 4,048 kbp) by Kamfwa et al. (2015a) is positioned between the last two in series of four phosphorus QTNs stretching between 3,864 and 4,076 kbp on the same chromosome from the present study. The explanatory power of QTNs from all studies is relatively low; there are just a few of them that could explain more than 10% of total phenotypic variability. The single exception is the study of Mahajan et al. (2017), who reported much higher explanatory power of the discovered QTLs, but is not comparable with the others, because it was based on different type of markers (SSRs).

Classical QTL analyses also yielded numerous marker-trait associations, which can also be used for comparison with GWAS findings, knowing the approximate positions of the QTLs. This is at least possible for meta-QTLs, thanks to their physical positions provided by Izquierdo et al. (2018). Three iron and zinc QTNs found by Katuuramu et al. (2018) fall into meta-QTL intervals on chromosomes 6 and 7, but for none of them there seem to be any candidate genes (reported by either group of authors). The explanatory power of meta-QTLs is stronger than for QTNs; they explain from 10 to 27% of total phenotypic variation probably because the mapping populations are derived from crosses between two homozygous parents.

The important aggravating factor for the detection and use of marker-trait associations is the fact that a lot of them are either environment or population specific. In studies that collected phenotypic data from more than one environment, most of the QTNs were environment-specific; for example, two iron QTNs detected in two different seasons by Diaz et al. (2020), or 3/7 (Ca) and 2/10 (Mn) QTNs detected in both years on both locations by Erdogmus et al. (2020). European collections of landraces (such as this Croatian collection) usually represent a mixture of genotypes of Andean and Mesoamerican origin and some inter-genepool hybrids. The strong effect of population structure can alter the effect of allele substitution to such proportion that it can be completely hidden at the whole population level, which can somehow be related to Blair and Izquierdo (2012) conclusion that QTLs can be genepool specific and therefore not detectable in inter-genepool crosses.

### Biofortification

Among the various possible strategies for use of QTNs in the biofortification breeding programs, Izquierdo et al. (2018) consider gene pyramidizing through marker-assisted selection too challenging, illustrating it by the example of stacking eight meta-QTL regions associated with both Fe and Zn in a single breeding line that has a probability of one in 256. They, therefore, suggest the genomic selection as the most promising strategy. Gains that could be achieved by allele substitution in the present study are smaller or larger than gains reported by other authors, depending on the mineral. Zn gains are larger than in Diaz et al. (2020) who reported a gain of 0.85 ppm, or Cichy et al. (2009) who reported gains of 0.6–1.5 ppm, and closer to Blair et al. (2009) who reported gains of 1.02–2.53 ppm. Ca and Mg gains are smaller than in Casañas et al. (2013) who reported gains of 0.85–11.40 g kg^–1^ for Ca and 0.33–0.40 g kg^–1^ for Mg, but for the dry weight of seed coat (in contrast to whole seed in present study). Blair et al. (2013) discuss the differences in concentrations of nutrients in seed coat and cotyledon, and conclude that they are due to different genes involved in mineral accumulation, as well as that through domestication accumulation of some nutrients shifted from seed coat to cotyledons. The achieved gains will not be fully utilized in human consumption, due to the presence of anti-nutrients, as well as due to losses during storage, processing and cooking. Besides the expected diminishing effect on the total content of nutrients, pre-soaking and cooking can as well increase the amount of bioavailable nutrients, due to a parallel decrease of the content of anti-nutrients (Fernandes et al., 2010). This is confirmed by Katuuramu et al. (2018), who analyzed mineral content of cooked beans and concluded that the results are in agreement with the studies on raw beans. Initial studies with already developed biofortified cultivars with twofold increased iron content (approximately from 50 to 100 ppm) showed only moderate increase in absorbed iron quantity, due to presence of higher levels of phytate (Petry et al., 2014; Tako et al., 2015). However, the consumption of biofortified beans can still significantly improve the iron status, as demonstrated in feeding trial (Haas et al., 2016). An alternative strategy for increasing the bioavailability of nutrients can be therefore the breeding for decreased anti-nutrient content, but just for areas with prevalent micronutrient deficiencies, while in others increased amounts of anti-nutrients like phytate might have beneficial effects on human health, reducing the risk of cancer and obesity (Blair et al., 2012).

## Data Availability Statement

The SNP dataset generated for this study is included in the Supplementary Table 2.

## Author Contributions

ZŠ and KC-S: conceptualization. JG and ZL: methodology. KC-S, BL, MV, MP, and ZL: investigation. JG and KC-S: writing-original draft preparation. ZŠ and ZL: writing – review and editing. All authors read and approved the final manuscript.

## Conflict of Interest

The authors declare that the research was conducted in the absence of any commercial or financial relationships that could be construed as a potential conflict of interest.

## Footnotes

1 https://www.diversityarrays.com/

## Supplementary Material

Supplementary Figure 1 Genetic structure of Croatian common bean accessions as estimated using Structure (Pritchard et al., 2000) at *K* = 2 and *K* = 3. Accessions are grouped according to phaseolin types (I, II, III). Each accession is represented by a column, and the color corresponds to the membership probability (i.e., *Q*-value) of the individual belonging to a particular cluster.

Supplementary Table 1 Common bean accession used in this study.

Supplementary Table 2 Information on SNP markers used in the study.

## Abbreviations

ALK: anaplastic lymphoma kinase
FDR: false discovery rate
GWAS: genome-wide association study
IBS: identity-by-state
LD: linkage disequilibrium
MAF: minor allele frequency
MLMM: multilocus mixed model
P3D: “population parameters previously determined”
SNP: single nucleotide polymorphism
QTL: quantitative trait locus
QTN: quantitative trait nucleotide.

## References

[B1] Astle W. Balding D. J. (2009). Population structure and cryptic relatedness in genetic association studies. *Stat. Sci.* 24 451–471. 10.1214/09-STS307

[B2] Ates D. Asciogul T. K. Nemli S. Erdogmus S. Esiyok D. Tanyolac M. B. (2018). Association mapping of days to flowering in common bean (*Phaseolus vulgaris* L.) revealed by DArT markers. *Mol. Breed.* 38 113. 10.1007/s11032-018-0868-0

[B3] Beebe S. Gonzalez A. V. Rengifo J. (2000). Research on trace minerals in the common bean. *Food Nutr. Bull.* 21 387–391. 10.1177/156482650002100408

[B4] Bellucci E. Bitocchi E. Rau D. Rodriguez M. Biagetti E. Giardini A. (2014). Genomics of origin, domestication and evolution of *Phaseolus vulgaris*. *Genomics Plant Genet. Resour.* 1 483–507. 10.1007/978-94-007-7572-5_20

[B5] Blair M. W. Astudillo C. Grusak M. A. Graham R. Beebe S. E. (2009). Inheritance of seed iron and zinc concentrations in common bean (*Phaseolus vulgaris* L.). *Mol. Breed.* 23 197–207. 10.1007/s11032-008-9225-z

[B6] Blair M. W. Astudillo C. Rengifo J. Beebe S. E. Graham R. (2011). QTL analyses for seed iron and zinc concentrations in an intra-genepool population of Andean common beans (*Phaseolus vulgaris* L.). *Theor. Appl. Genet.* 122 511–521. 10.1007/s00122-010-1465-8 21113704

[B7] Blair M. W. Herrera A. L. Sandoval T. H. Caldas G. V. Filleppi M. Sparvoli F. (2012). Inheritance of seed phytate and phosphorus levels in common bean (*Phaseolus vulgaris* L.) and association with newly-mapped candidate genes. *Mol. Breed.* 30 1265–1277. 10.1007/s11032-012-9713-z

[B8] Blair M. W. Izquierdo P. (2012). Use of the advanced backcross-QTL method to transfer seed mineral accumulation nutrition traits from wild to Andean cultivated common beans. *Theor. Appl. Genet.* 125 1015–1031. 10.1007/s00122-012-1891-x 22718301

[B9] Blair M. W. Izquierdo P. Astudillo C. Grusak M. A. (2013). A legume biofortification quandary: variability and genetic control of seed coat micronutrient accumulation in common beans. *Front. Plant Sci.* 4:275. 10.3389/fpls.2013.00275 23908660 PMC3725406

[B10] Blair M. W. Medina J. I. Astudillo C. Rengifo J. Beebe S. E. Machado G. (2010). QTL for seed iron and zinc concentration and content in a Mesoamerican common bean (*Phaseolus vulgaris* L.) population. *Theor. Appl. Genet.* 121 1059–1070. 10.1007/s00122-010-1371-0 20532862

[B11] Bradbury P. J. Zhang Z. Kroon D. E. Casstevens T. M. Ramdoss Y. Buckler E. S. (2007). TASSEL: Software for association mapping of complex traits in diverse samples. *Bioinformatics* 23 2633–2635. 10.1093/bioinformatics/btm308 17586829

[B12] Browning B. L. Zhou Y. Browning S. R. (2018). A one-penny imputed genome from next-generation reference panels. *Am. J. Hum. Genet.* 103 338–348. 10.1016/j.ajhg.2018.07.015 30100085 PMC6128308

[B13] Câmara C. R. S. Urrea C. A. Schlegel V. (2013). Pinto beans (*Phaseolus vulgaris* L.) as a functional food: implications on human health. *Agriculture* 3 90–111. 10.3390/agriculture3010090

[B14] Carović-Stanko K. Liber Z. Vidak M. Barešić A. Grdiša M. Lazarević B. (2017). Genetic diversity of croatian common bean landraces. *Front. Plant Sci.* 8:604. 10.3389/fpls.2017.00604 28473842 PMC5397504

[B15] Casañas F. Pérez-Vega E. Almirall A. Plans M. Sabaté J. Ferreira J. J. (2013). Mapping of QTL associated with seed chemical content in a RIL population of common bean (*Phaseolus vulgaris* L.). *Euphytica* 192 279–288. 10.1007/s10681-013-0880-8

[B16] Chávez-Servia J. L. Heredia-García E. Mayek-Pérez N. Aquino-Bolaños E. N. Hernández-Delgado S. Carrillo-Rodríguez J. C. (2016). “Diversity of common bean (*Phaseolus vulgaris* L.) landraces and the nutritional value of their grains,” in *Grain Legumes*, ed. Goyal A. (London: IntechOpen), 10.5772/63439

[B17] Cichy K. A. Caldas G. V. Snapp S. S. Blair M. W. (2009). QTL analysis of seed iron, zinc, and phosphorus levels in an andean bean population. *Crop Sci.* 128 1555–1567. 10.2135/cropsci2008.10.0605

[B18] Cichy K. A. Wiesinger J. A. Mendoza F. A. (2015). Genetic diversity and genome-wide association analysis of cooking time in dry bean (*Phaseolus vulgaris* L.). *Theor. Appl. Genet.* 128 1555–1567. 10.1007/s00122-015-2531-z 26003191

[B19] Dahl A. Iotchkova V. Baud A. Johansson S. Gyllensten U. Soranzo N. (2016). A multiple-phenotype imputation method for genetic studies. *Nat. Genet.* 48 466–472. 10.1038/ng.3513 26901065 PMC4817234

[B20] Diaz S. Ariza-Suarez D. Izquierdo P. Lobaton J. D. de la Hoz J. F. Acevedo F. (2020). Genetic mapping for agronomic traits in a MAGIC population of common bean (*Phaseolus vulgaris* L.) under drought conditions. *BMC Genomics* 21:799. 10.1186/s12864-020-07213-6 PMC7670608 33198642

[B21] Diepenbrock C. H. Gore M. A. (2015). Closing the divide between human nutrition and plant breeding. *Crop Sci.* 55 1437–1448. 10.2135/cropsci2014.08.0555

[B22] Diniz W. J. S. Mazzoni G. Coutinho L. L. Banerjee P. Geistlinger L. Cesar A. S. M. (2019). Detection of co-expressed pathway modules associated with mineral concentration and meat quality in nelore cattle. *Front. Genet.* 10:210. 10.3389/fgene.2019.00210 30930938 PMC6424907

[B23] Endelman J. B. Jannink J. L. (2012). Shrinkage estimation of the realized relationship matrix. *G3 Genes Genomes Genet.* 2 1405–1413. 10.1534/g3.112.004259 23173092 PMC3484671

[B24] Erdogmus S. Ates D. Nemli S. Yagmur B. Asciogul T. K. Ozkuru E. (2020). Genome-wide association studies of Ca and Mn in the seeds of the common bean (*Phaseolus vulgaris* L.). *Genomics* 112 4536–4546. 10.1016/j.ygeno.2020.03.030 32763354

[B25] Fernandes A. C. Nishida W. da Costa Proença R. P. (2010). Influence of soaking on the nutritional quality of common beans (*Phaseolus vulgaris* L.) cooked with or without the soaking water: a review. *Int. J. Food Sci. Technol.* 45 2209–2218. 10.1111/j.1365-2621.2010.02395.x

[B26] Fritsche-Neto R. De Souza T. L. P. O. Pereira H. S. De Faria L. C. Melo L. C. Novaes E. (2019). Association mapping in common bean revealed regions associated with anthracnose and angular leaf spot resistance. *Sci. Agric.* 76 321–327. 10.1590/1678-992X-2017-0306

[B27] Galeano C. H. Cortés A. J. Fernández A. C. Soler Á Franco-Herrera N. Makunde G. (2012). Gene-based single nucleotide polymorphism markers for genetic and association mapping in common bean. *BMC Genet.* 13:48. 10.1186/1471-2156-13-48 22734675 PMC3464600

[B28] Gianola D. Fariello M. I. Naya H. Schön C. C. (2016). Genome-wide association studies with a genomic relationship matrix: a case study with wheat and arabidopsis. *G3 Genes Genomes Genet.* 6 3241–3256. 10.1534/g3.116.034256 27520956 PMC5068945

[B29] Gioia T. Logozzo G. Attene G. Bellucci E. Benedettelli S. Negri V. (2013). Evidence for introduction bottleneck and extensive inter-gene pool (Mesoamerica x Andes) hybridization in the European common bean (*Phaseolus vulgaris* L.) Germplasm. *PLoS One* 8:e75974. 10.1371/journal.pone.0075974 24098412 PMC3788063

[B30] Goretti D. Bitocchi E. Bellucci E. Rodriguez M. Rau D. Gioia T. (2014). Development of single nucleotide polymorphisms in *Phaseolus vulgaris* and related *Phaseolus* spp. *Mol. Breed.* 33 531–544. 10.1007/s11032-013-9970-5

[B31] Gouveia C. S. S. Freitas G. Brito J. H. de Slaski J. J. de Carvalho M. ÂA. P. (2014). Nutritional and mineral variability in 52 accessions of common bean varieties (*Phaseolus vulgaris* L.) from Madeira Island. *Agric. Sci.* 5 317–329. 10.4236/as.2014.54034

[B32] Haas J. D. Luna S. V. Lung’aho M. G. Wenger M. J. Murray-Kolb L. E. Beebe S. (2016). Consuming iron biofortified beans increases iron status in rwandan women after 128 days in a randomized controlled feeding trial. *J. Nutr.* 46 1586–1592. 10.3945/jn.115.224741 27358417

[B33] Hill W. G. Weir B. S. (1988). Variances and covariances of squared linkage disequilibria in finite populations. *Theor. Popul. Biol.* 33 54–78. 10.1016/0040-5809(88)90004-4 3376052

[B34] Hirschi K. D. (2009). Nutrient biofortification of food crops. *Annu. Rev. Nutr.* 29 401–421. 10.1146/annurev-nutr-080508-141143 19400753

[B35] Hoyos-Villegas V. Song Q. Kelly J. D. (2017). Genome-wide association analysis for drought tolerance and associated traits in common bean. *Plant Genome* 10 1–17. 10.3835/plantgenome2015.12.0122 28464065

[B36] Islam F. M. A. Basford K. E. Jara C. Redden R. J. Beebe S. (2002). Seed compositional and disease resistance differences among gene pools in cultivated common bean. *Genet. Resour. Crop Evol.* 49 285–293. 10.1023/A:1015510428026

[B37] Izquierdo P. Astudillo C. Blair M. W. Iqbal A. M. Raatz B. Cichy K. A. (2018). Meta-QTL analysis of seed iron and zinc concentration and content in common bean (*Phaseolus vulgaris* L.). *Theor. App. Gen.* 131 1645–1658. 10.1007/s00122-018-3104-8 29752522

[B38] Jaccoud D. Peng K. Feinstein D. Kilian A. (2001). Diversity arrays: a solid state technology for sequence information independent genotyping. *Nucleic Acids Res.* 29:E25. 10.1093/nar/29.4.e25 11160945 PMC29632

[B39] Kamfwa K. Cichy K. A. Kelly J. D. (2015a). Genome-wide association analysis of symbiotic nitrogen fixation in common bean. *Theor. Appl. Genet.* 128 1999–2017. 10.1007/s00122-015-2562-5 26133733

[B40] Kamfwa K. Cichy K. A. Kelly J. D. (2015b). Genome-Wide association study of agronomic traits in common bean. *Plant Genome* 8:plantgenome2014.09.0059. 10.3835/plantgenome2014.09.0059 33228312

[B41] Kami J. Velásquez V. B. Debouck D. G. Gepts P. (1995). Identification of presumed ancestral DNA sequences of phaseolin in *Phaseolus vulgaris*. *Proc. Natl. Acad. Sci. U.S.A.* 92 1101–1104. 10.1073/pnas.92.4.1101 7862642 PMC42645

[B42] Katuuramu D. N. Hart J. P. Porch T. G. Grusak M. A. Glahn R. P. Cichy K. A. (2018). Genome-wide association analysis of nutritional composition-related traits and iron bioavailability in cooked dry beans (*Phaseolus vulgaris* L.). *Mol. Breed.* 38:44. 10.1007/s11032-018-0798-x

[B43] Leitão S. T. Dinis M. Veloso M. M. Šatović Z. Vaz Patto M. C. (2017). Establishing the bases for introducing the unexplored portuguese common bean germplasm into the breeding world. *Front. Plant Sci.* 8:1296. 10.3389/fpls.2017.01296 28798757 PMC5526916

[B44] Lipka A. E. Tian F. Wang Q. Peiffer J. Li M. Bradbury P. J. (2012). GAPIT: Genome association and prediction integrated tool. *Bioinformatics* 28 2397–2399. 10.1093/bioinformatics/bts444 22796960

[B45] Mahajan R. Zargar S. M. Salgotra R. K. Singh R. Wani A. A. Nazir M. (2017). Linkage disequilibrium based association mapping of micronutrients in common bean (*Phaseolus vulgaris* L.): a collection of Jammu & Kashmir. *India*. *3 Biotech* 7:295. 10.1007/s13205-017-0928-x 28868222 PMC5577372

[B46] Mangin B. Siberchicot A. Nicolas S. Doligez A. This P. Cierco-Ayrolles C. (2012). Novel measures of linkage disequilibrium that correct the bias due to population structure and relatedness. *Heredity (Edinb)* 108 285–291. 10.1038/hdy.2011.73 21878986 PMC3282397

[B47] Maras M. Pipan B. Šuštar-Vozliè J. Todorović V. Ðurić G. Vasić M. (2015). Examination of genetic diversity of common bean from the western Balkans. *J. Am. Soc. Hortic. Sci.* 140 308–316. 10.21273/jashs.140.4.308

[B48] Moghaddam S. M. Mamidi S. Osorno J. M. Lee R. Brick M. Kelly J. (2016). Genome-Wide association study identifies candidate loci underlying agronomic traits in a Middle American diversity panel of common bean. *Plant Genome* 9 1–21. 10.3835/plantgenome2016.02.0012 27902795

[B49] Muñoz P. R. Resende M. F. R. Gezan S. A. Resende M. D. V. de los Campos G. Kirst M. (2014). Unraveling additive from nonadditive effects using genomic relationship matrices. *Genetics* 198 1759–1768. 10.1534/genetics.114.171322 25324160 PMC4256785

[B50] Muqaddasi Q. H. Reif J. C. Li Z. Basnet B. R. Dreisigacker S. Röder M. S. (2017). Genome-wide association mapping and genome-wide prediction of anther extrusion in CIMMYT spring wheat. *Euphytica* 213:73. 10.1007/s10681-017-1863-y

[B51] Myers J. R. Wallace L. T. Moghaddam S. M. Kleintop A. E. Echeverria D. Thompson H. J. (2019). Improving the health benefits of snap bean: genome-wide association studies of total phenolic content. *Nutrients* 11:2509. 10.3390/nu11102509 31635241 PMC6835575

[B52] Nascimento M. Nascimento A. C. C. Silva F. F. E. Barili L. D. Do Vale N. M. Carneiro J. E. (2018). Quantile regression for genome-wide association study of flowering time-related traits in common bean. *PLoS One* 13:e190303. 10.1371/journal.pone.0190303 29300788 PMC5754186

[B53] Nemli S. Asciogul T. K. Kaya H. B. Kahraman A. Eşiyok D. Tanyolac B. (2014). Association mapping for five agronomic traits in the common bean (*Phaseolus vulgaris* L.). *J. Sci. Food Agric.* 41 389–404. 10.1002/jsfa.6664 24659306

[B54] Nemli S. Kaygisiz Aşçioğul T. Ateç D. Eşıyok D. Tanyolaç M. B. (2017). Diversity and genetic analysis through DArTseq in common bean (*Phaseolus vulgaris* L.) germplasm from Turkey. *Turkish J. Agric. For.* 94 3141–3151. 10.3906/tar-1707-89 31411186

[B55] Novakazi F. Afanasenko O. Anisimova A. Platz G. J. Snowdon R. Kovaleva O. (2019). Genetic analysis of a worldwide barley collection for resistance to net form of net blotch disease (*Pyrenophora teres* f. *teres*). *Theor. Appl. Genet.* 132 2633–2650. 10.1007/s00122-019-03378-1 31209538

[B56] Nwadike C. Okere A. Nwosu D. Okoye C. Vange T. Apuyor B. (2018). Proximate and nutrient composition of some common bean (*Phaseolus vulgaris* L.) and Cowpea (*Vigna unguiculata* L. Walp.) accessions of Jos- Plateau, Nigeria. *J. Agric. Ecol. Res. Int.* 15 1–9. 10.9734/jaeri/2018/42138

[B57] Palèić I. Karažija T. Petek M. Lazarević B. Herak Ćustić M. Gunjača J. (2018). Relationship between origin and nutrient content of croatian common bean landraces. *J. Cent. Eur. Agric.* 19 490–502. 10.5513/JCEA01/19.3.2103

[B58] Perseguini J. M. K. C. Oblessuc P. R. Rosa J. R. B. F. Gomes K. A. Chiorato A. F. Carbonell S. A. M. (2016). Genome-wide association studies of anthracnose and angular leaf spot resistance in common bean (*Phaseolus vulgaris* L.). *PLoS One* 11:e150506. 10.1371/journal.pone.0150506 26930078 PMC4773255

[B59] Petry N. Egli I. Gahutu J. B. Tugirimana P. L. Boy E. Hurrell R. (2014). Phytic acid concentration influences iron bioavailability from biofortified beans in rwandese women with low iron status. *J. Nutr.* 144 1681–1687. 10.3945/jn.114.192989 25332466

[B60] Pfeiffer W. H. McClafferty B. (2007). HarvestPlus: breeding crops for better nutrition. *Crop Sci.* 47 88–105. 10.2135/cropsci2007.09.0020IPBS

[B61] Pritchard J. K. Stephens M. Donnelly P. (2000). Inference of population structure using multilocus genotype data. *Genetics* 155 945–959. 10835412 10.1093/genetics/155.2.945 PMC1461096

[B62] Rau D. Murgia M. L. Rodriguez M. Bitocchi E. Bellucci E. Fois D. (2019). Genomic dissection of pod shattering in common bean: mutations at non-orthologous loci at the basis of convergent phenotypic evolution under domestication of leguminous species. *Plant J.* 97 693–714. 10.1111/tpj.14155 30422331

[B63] Razvi S. M. Dar M. H. Groach R. Bhat A. (2017). Molecular diversity and gene pool structure in common bean (*Phaseolus vulgaris* L.): a review. *Int. J. Curr. Trends Sci. Technol.* 7 20185–20202. 10.15520/ctst.v7i11.93

[B64] Resende R. T. de Resende M. D. V. Azevedo C. F. Silva F. F. E. Melo L. C. Pereira H. S. (2018). Genome-wide association and Regional Heritability Mapping of plant architecture, lodging and productivity in phaseolus vulgaris. *G3 Genes Genomes Genet.* 8 2841–2854. 10.1534/g3.118.200493 29967054 PMC6071601

[B65] Ribeiro N. D. Maziero S. M. Prigol M. Nogueira C. W. Rosa D. P. Possobom M. T. D. F. (2012). Mineral concentrations in the embryo and seed coat of common bean cultivars. *J. Food Compos. Anal.* 26 89–95. 10.1016/j.jfca.2012.03.003

[B66] Schmutz J. McClean P. E. Mamidi S. Wu G. A. Cannon S. B. Grimwood J. (2014). A reference genome for common bean and genome-wide analysis of dual domestications. *Nat. Genet.* 46 707–713. 10.1038/ng.3008 24908249 PMC7048698

[B67] Segura V. Vilhjálmsson B. J. Platt A. Korte A. Seren Ü Long Q. (2012). An efficient multi-locus mixed-model approach for genome-wide association studies in structured populations. *Nat. Genet.* 44 825–830. 10.1038/ng.2314 22706313 PMC3386481

[B68] Semba R. D. (2016). The rise and fall of protein malnutrition in global health. *Ann. Nutr. Metab*. 69 79–88. 10.1159/000449175 27576545 PMC5114156

[B69] Shi C. Navabi A. Yu K. (2011). Association mapping of common bacterial blight resistance QTL in Ontario bean breeding populations. *BMC Plant Biol.* 11:52. 10.1186/1471-2229-11-52 21435233 PMC3078875

[B70] Soltani A. Mafimoghaddam S. Oladzad-Abbasabadi A. Walter K. Kearns P. J. Vasquez-Guzman J. (2018). Genetic analysis of flooding tolerance in an andean diversity panel of dry bean (*Phaseolus vulgaris* L.). *Front. Plant Sci.* 9:767. 10.3389/fpls.2018.00767 29928287 PMC5997968

[B71] Soltani A. MafiMoghaddam S. Walter K. Restrepo-Montoya D. Mamidi S. Schroder S. (2017). Genetic architecture of flooding tolerance in the dry bean middle-American diversity panel. *Front. Plant Sci.* 8:1183. 10.3389/fpls.2017.01183 28729876 PMC5498472

[B72] Storey J. D. Bass A. J. Dabney A. Robinson D. (2020). *qvalue: Q-Value Estimation for False Discovery Rate Control Version 2.22.0 from Bioconductor.* Available online at: https://rdrr.io/bioc/qvalue/ (accessed November 25, 2020).

[B73] Storey J. D. Tibshirani R. (2003). Statistical significance for genomewide studies. *Proc. Natl. Acad. Sci. U.S.A.* 100 9440–9445. 10.1073/pnas.1530509100 12883005 PMC170937

[B74] Tako E. Reed S. Anandaraman A. Beebe S. E. Hart J. J. Glahn R. P. (2015). Studies of cream seeded carioca beans (*Phaseolus vulgaris* L.) from a Rwandan efficacy trial: *In Vitro* and *In Vivo* screening tools reflect human studies and predict beneficial results from iron biofortified beans. *PLoS One* 10:e138479. 10.1371/journal.pone.0138479 PMC4575050 26381264

[B75] Tapiero H. Townsend D. M. Tew K. D. (2003). Trace elements in human physiology and pathology. *Copper. Biomed. Pharmacother.* 57 386–398. 10.1016/S0753-3322(03)00012-X 14652164 PMC6361146

[B76] Tock A. J. Fourie D. Walley P. G. Holub E. B. Soler A. Cichy K. A. (2017). Genome-wide linkage and association mapping of halo blight resistance in common bean to race 6 of the globally important bacterial pathogen. *Front. Plant Sci.* 8:1170. 10.3389/fpls.2017.01170 28736566 PMC5500643

[B77] Valdisser P. A. M. R. Pereira W. J. Almeida Filho J. E. Müller B. S. F. Coelho G. R. C. de Menezes I. P. P. (2017). In-depth genome characterization of a Brazilian common bean core collection using DArTseq high-density SNP genotyping. *BMC Genomics* 18:423. 10.1186/s12864-017-3805-4 28558696 PMC5450071

[B78] Villordo-Pineda E. González-Chavira M. M. Giraldo-Carbajo P. Acosta-Gallegos J. A. Caballero-Pérez J. (2015). Identification of novel drought-tolerant-associated SNPs in common bean (*Phaseolus vulgaris*). *Front. Plant Sci.* 6:546. 10.3389/fpls.2015.00546 26257755 PMC4508514

[B79] Wenzl P. Carling J. Kudrna D. Jaccoud D. Huttner E. Kleinhofs A. (2004). Diversity Arrays Technology (DArT) for whole-genome profiling of barley. *Proc. Natl. Acad. Sci. U.S.A.* 101 9915–9920. 10.1073/pnas.0401076101 15192146 PMC470773

[B80] Yang J. Lee S. H. Goddard M. E. Visscher P. M. (2011). GCTA: a tool for genome-wide complex trait analysis. *Am. J. Hum. Genet.* 88 76–82. 10.1016/j.ajhg.2010.11.011 21167468 PMC3014363

[B81] Yeken M. Z. Akpolat H. Karaköy T. Çiftçi V. (2018). Assessment of mineral content variations for biofortification of the bean seed. *Uluslararası Tarım ve Yaban Hayatı Bilim. Derg.* 4 261–269. 10.24180/ijaws.455311

[B82] Yin L. Zhang H. Tang Z. Xu J. Yin D. Yuan X. (2020). rMVP: a memory-efficient, Visualization-enhanced, and parallel-1 accelerated tool for genome-wide association study. *bioRxiv [Preprint]* 10.1101/2020.08.20.258491 PMC9040015 33662620

[B83] Yu J. Pressoir G. Briggs W. H. Bi I. V. Yamasaki M. Doebley J. F. (2006). A unified mixed-model method for association mapping that accounts for multiple levels of relatedness. *Nat. Genet.* 38 203–208. 10.1038/ng1702 16380716

[B84] Zhang Z. Ersoz E. Lai C. Q. Todhunter R. J. Tiwari H. K. Gore M. A. (2010). Mixed linear model approach adapted for genome-wide association studies. *Nat. Genet.* 42 355–360. 10.1038/ng.546 20208535 PMC2931336

[B85] Zhang Z. Schwartz S. Wagner L. Miller W. (2000). A greedy algorithm for aligning DNA sequences. *J. Comput. Biol.* 7 203–214. 10.1089/10665270050081478 10890397

[B86] Zhu Z. Bakshi A. Vinkhuyzen A. A. E. Hemani G. Lee S. H. Nolte I. M. (2015). Dominance genetic variation contributes little to the missing heritability for human complex traits. *Am. J. Hum. Genet.* 96 377–385. 10.1016/j.ajhg.2015.01.001 25683123 PMC4375616

[B87] Zuiderveen G. H. Padder B. A. Kamfwa K. Song Q. Kelly J. D. (2016). Genome-Wide association study of anthracnose resistance in andean beans (*Phaseolus vulgaris*). *PLoS One* 11:e156391. 10.1371/journal.pone.0156391 27270627 PMC4894742

