# Supplementary figures and images for: Genome-Wide Association Studies of Mineral Content in Common Bean
Source: Front Plant Sci. 2021 Mar 5;12:636484. doi: 10.3389/fpls.2021.636484 (PMC7982862; doi:10.3389/fpls.2021.636484)

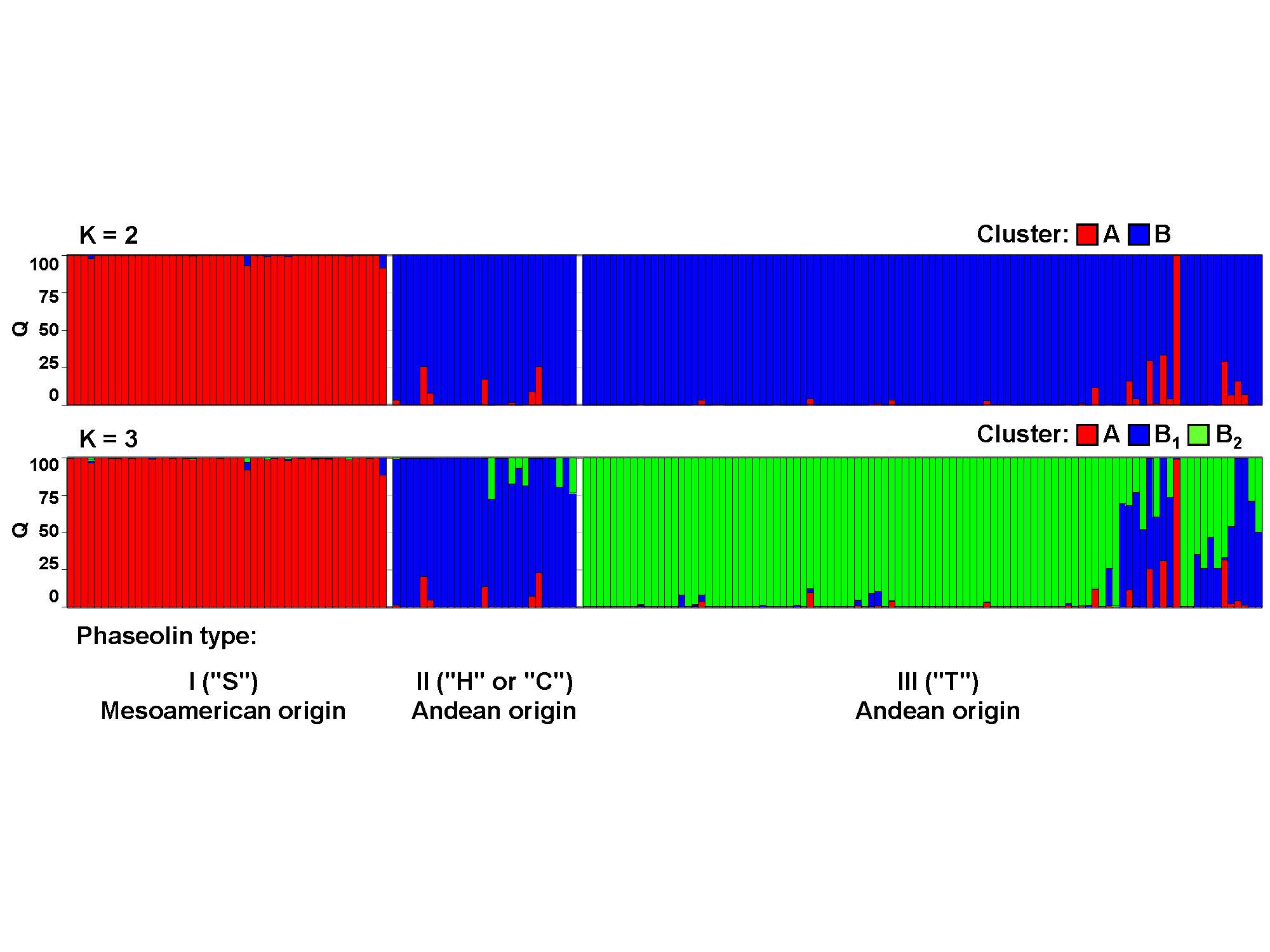

Supplement: Supplementary Figure 1 — Genetic structure of Croatian common bean accessions as estimated using Structure (Pritchard et al., 2000) at K = 2 and K = 3. Accessions are grouped according to phaseolin types (I, II, III). Each accession is represented by a column, and the color corresponds to the membership probability (i.e., Q-value) of the individual belonging to a particular cluster. [file Image_1.JPEG]
